# Sclerosing mucoepidermoid carcinoma of salivary glands

**DOI:** 10.1007/s00428-024-03970-x

**Published:** 2024-11-14

**Authors:** Bacem Khalele Othman, Martina Bradová, Roderick H. W. Simpson, Jan Laco, Abbas Agaimy, Miguel Rito, Stephan Ihrler, Petr Steiner, Petr Grossmann, Veronika Hájková, Gisele de Rezende, Montse Goma, Senada Koljenovic, Isabel Fonseca, Michal Michal, Ilmo Leivo, Alena Skalova

**Affiliations:** 1 Department of Pathology, Faculty of Medicine in Pilsen, Charles University, Pilsen, Czech Republic; 2 Bioptic Laboratory, Ltd, Pilsen, Czech Republic; 3 Department of Pathology, University of Calgary, Calgary, Alberta Canada; 4 The Fingerland Department of Pathology, Faculty of Medicine, Charles University, Hradec Králové and University Hospital Hradec Králové, Hradec Králové, Czech Republic; 5 Institute of Pathology, University Hospital Erlangen, Friedrich‐Alexander University Erlangen‐Nürnberg (FAU), Comprehensive Cancer Center (CCC) Erlangen-EMN, Erlangen, Germany; 6 Department of Pathology, Faculdade de Medicina, Instituto Português de Oncologia de Lisboa Francisco Gentil & Institute of Pathology, Universidade de Lisboa, Lisbon, Portugal; 7 DERMPATH, Munich, Germany; 8 Molecular and Genetic Laboratory, Bioptic Laboratory, Ltd, Pilsen, Czech Republic; 9 Department of Anatomic Histopathology and Cytogenetics, Department of Laboratory Medicine, Niguarda Cancer Center, Milan, Italy; 10 Department of Pathology, Hospital Universitari de Bellvitge, Barcelona, Spain; 11 Department of Pathology, Antwerp University Hospital, University of Antwerp, Antwerp, Belgium; 12 Department of Pathology, Erasmus University Medical Center, Rotterdam, The Netherlands; 13 Institute of Biomedicine, Pathology, University of Turku and Department of Pathology, Turku University Hospital, Turku, Finland

**Keywords:** Sclerosing mucoepidermoid carcinoma, Salivary gland, *MAML2* rearrangement, Sclerosis, Tissue eosinophilia, Keloid-like stromal fibrosis, IgG4, SMEC

## Abstract

Sclerosing mucoepidermoid carcinoma (SMEC) of the salivary glands is a rare variant of low-grade mucoepidermoid carcinoma with scanty cellular atypia characterized by marked fibrosis/sclerosis and a rich inflammatory infiltrate. Herein, we report 25 unpublished cases of SMEC, two of them with prominent eosinophilia (2/25; 8%) and three with abundant IgG4-positive plasma cells (3/25; 12%). In our series of salivary SMEC, molecular analysis using fluorescence in situ hybridization (FISH) and/or next-generation sequencing (NGS) provided evidence of *MAML2* gene rearrangement in 18 cases of the 21 analyzable cases tested (86%), while this gene locus was intact in 3 cases (14%). This study focuses on the diagnostic criteria of salivary SMEC given its challenge of abundant collagenous stroma, minimal residual neoplastic areas, and inconspicuous mucous cells. Follow-up data of our cases indicate that salivary SMECs have favorable outcomes. Molecular analysis for *MAML2* gene rearrangement suggests that SMECs of salivary glands represent a rare variant of conventional low-grade MECs of salivary glands. In contrast, SMECs of the thyroid gland are genetically distinct from salivary-type thyroid MECs.

**Supplementary Information:**

The online version contains supplementary material available at 10.1007/s00428-024-03970-x.

## Introduction

Mucoepidermoid carcinoma (MEC) is the most common malignant tumor of major and minor salivary glands characterized by mucous, intermediate, and epidermoid (squamoid) tumor cells forming cystic and solid growth patterns and usually associated with *MAML2* rearrangement [1]. Diagnosis of conventional MEC is generally straightforward on histologic grounds alone. In less typical cases, the application of mucin histochemistry (mucicarmine and/or Alcian Blue/PAS/ and/or PAS-diastase stain) facilitates the identification of true intracytoplasmic mucin.

Classical MEC is in most instances an easily recognizable tumor. There is, however, a spectrum of rare histologic variants of MEC, such as oncocytic [2, 3]_,_ Warthin-like [4, 5], ciliated [4], clear cell [6, 7], pigmented [8], spindle cell [8–10], and mucoacinar [11] that differ from the conventional appearance. In addition, the availability of molecular testing has also made it possible to describe MEC devoid of squamoid cells using immunohistochemistry [12], monomorphic MEC with a pure spindle and clear cell pattern [13], and MEC with a unique trabecular growth pattern [14], thus expanding the histologic and immunohistochemical spectrum of *MAML2*-rearranged salivary gland tumors. In such cases, reaching the correct diagnosis on histological grounds alone can be difficult. Such histological variants of MEC have now been confirmed to have characteristic molecular alterations involving the fusion transcripts *CRTC1::MAML2* or *CRTC3::MAML2.*

Molecular testing may also be crucial for an accurate diagnosis of sclerosing mucoepidermoid carcinoma (SMEC), a rare and diagnostically challenging variant of salivary MEC. Based on previously described cases, SMEC has a tendency to form keloid-like sclerotic stroma and foci of densely packed inflammatory infiltrates situated subcapsularly or within the neoplasm itself [15, 16]. These inflammatory infiltrates, containing eosinophils, plasma cells, and/or lymphocytes, are usually intermingled with solid neoplastic nests [17–19]. In cases with prevalent eosinophils in the inflammatory infiltrate, the designation of salivary sclerosing mucoepidermoid carcinoma with eosinophilia (SMECE) has been used in the literature [20, 21].

Accurate diagnostic criteria of SMEC, however, remain controversial. Simple FISH analysis to provide evidence of *MAML2* gene rearrangement may sometimes fail. Given such challenges, it is urgent to search for diagnostic histological criteria of SMEC in order to prevent misdiagnoses in difficult cases, particularly if molecular testing is not available.

It has been speculated that salivary SMECE closely resembles the thyroid counterpart, which is now included in the WHO Classification as a distinct entity [22]. Thyroid SMECE is a thyroid carcinoma composed of epidermoid and mucous cells in a background of marked stromal sclerosis with infiltration of eosinophils and lymphocytes. Genomic studies of thyroid SMECE have so far been largely uninformative, showing a notable absence of *MAML2* rearrangements characteristic of mucoepidermoid carcinoma or *BRAF* mutations [23–25]. Salivary SMECE [20, 21] has sparked a major controversy posing the question if it represents an equivalent of thyroid SMECE [22].

The aim of this study is to document 25 new cases of salivary SMEC/SMECE from consult files, including histomorphological and immunohistochemical features, molecular testing, and clinical outcomes when available. There are no reliable published criteria for the proportion of sclerosis and the composition of inflammatory infiltrates required for the diagnosis of salivary SMEC. Based on the study of our cases and review of the literature, we propose a set of major criteria for diagnosing SMEC, including keloid-like sclerosis representing more than 50% of the tumor volume and an associated heterogenous inflammatory infiltrate at the periphery and within the tumor itself. The frequency of salivary SMECs is difficult to assess, being no more than 5% of all MECs, while it may be much lower than that, as SMEC tends to be over-represented in consultation practices. In addition, we address the question of the similarity between salivary and thyroid SMEC/SMECE. Our cohort represents the largest series of salivary SMECs so far.

## Materials and methods

A retrospective search in the authors’ registries was conducted to identify MECs characterized by remarkable keloid-like sclerotic stroma and dense inflammatory infiltrate. In total, 25 cases of SMEC were retrieved from the consultation files of the Tumor Registry at the Department of Pathology, Faculty of Medicine in Pilsen and Bioptic Laboratory, Ltd. in Pilsen, Czech Republic, and tumor registries of the co-authors. All cases were reviewed by the senior author (AS) and three other head and neck pathologists (RHWS, BK, and MB), and it was confirmed that they met the diagnostic criteria of SMEC, in particular, keloid-like sclerosis in more than 50% of the total tumor volume and abundant variable inflammatory infiltrate, in particular, rich on lymphocytes, plasma cells, and/or eosinophils.

Clinical features and outcomes (e.g., age, sex, site of primary tumor, follow-up period, recurrence, and distant metastasis) were recorded. Clinical information on the cases was collected from hospital records and the referring pathologists. The study was approved by the institutional review board.

### Histology and immunohistochemistry

For conventional microscopy, tissues were fixed in formalin, processed routinely, embedded in paraffin (FFPE), cut, and stained with hematoxylin and eosin.

For immunohistochemistry, 4-μm-thick sections were cut from the paraffin blocks and mounted on positively charged slides (TOMO, Matsunami Glass INC, Osaka, Japan). Sections were processed on a BenchMark ULTRA (Ventana Medical Systems, Tucson, AZ), deparaffinized, and subjected to heat-induced epitope retrieval by immersion in CC1 solution (pH 8.6) at 95 °C. All primary antibodies used in this study are summarized in Table 1. Visualization was performed using the ultraView Universal DAB Detection Kit (Roche, Tucson, AZ) and the ultraView Universal Alkaline Phosphatase Red Detection Kit (Roche, Tucson, AZ). The slides were counterstained with Mayer’s hematoxylin. Appropriate positive and negative controls were employed.

**Table 1:** Antibodies used for immunohistochemical study

Antibody specificity	Clone	Dilution	Antigen retrieval/time	Source
AE1/3	AE1/AE3 + PCK26	RTU	EnVision high pH /30 min	Dako
CK7	OV-TL 12/30	1:800	EnVision high pH/30 min	Dako
CK14	SP53	1:800	EnVision high pH/30 min	Cell Marque
p63	DAK-p63	RTU	EnVision low pH/30 min	Dako
p40	DAK-p40	RTU	EnVision low pH/30 min	Dako
SOX 10	SP267	RTU	CC1/64 min	Cell Marque
Ki-67	MIB-1	RTU	EnVision low pH/30 min	Dako
IgG	polyclonal	RTU	CC1 36 min	Cell Marque
IgG4	HP6025	1:800	CC1 64 min	Invitrogen

*RTU* ready to use; *CC1*, EDTA buffer pH 8.6 at 95 °C, EnVision high pH 9.0 at 97 °C, EnVision low pH 6.0 at 97 °C; *min* minutes

### Molecular genetic studies

#### Next-generation sequencing

The in-house customized version of Archer FusionPlex Solid Kit was used to construct a cDNA library for detecting fusion transcripts in 118 genes (including *AKT1*, *AKT3*, *ALK*, *AR*, *ARHGAP26*, *AXL*, *BCOR*, *BRAF*, *BRD3*, *BRD4*, *CALCA*, *CAMTA1*, *CCNB3*, *CCND1*, *CD274*, *CIC*, *CSF1*, *CSF1R*, *DNAJB1*, *EGFR*, *EPC1*, *ERBB2*, *ERBB4*, *ERG*, *ESR1*, *ESRRA*, *ETV1*, *ETV4*, *ETV5*, *ETV6*, *EWSR1*, *FGFR1*, *FGFR2*, *FGFR3*, *FGR*, *FOXO1*, *FOXO4*, *FUS*, *GLI1*, *GNAS*, *GPI*, *GRB7*, *HMGA2*, *CHMP2A*, *INSR*, *JAK2*, *JAK3*, *JAZF1*, *KRT20*, *KRT7*, *MAML2*, *MAP3K3*, *MAP3K8*, *MAST1*, *MAST2*, *MEAF6*, *MET*, *MGEA5*, *MKL2*, *MN1*, *MSMB*, *MUSK*, *MYB*, *MYBL1*, *MYOD1*, *NCOA1*, *NCOA2*, *NOTCH1*, *NOTCH2*, *NR4A3*, *NRG1*, *NTRK1*, *NTRK2*, *NTRK3*, *NUMBL*, *NUTM1*, *PAX3*, *PDGFB*, *PDGFRA*, *PDGFRB*, *PHF1*, *PIK3CA*, *PKN1*, *PLAG1*, *PPARG*, *PRKACA*, *PRKACB*, *PRKCA*, *PRKCB*, *PRKD1*, *PRKD2*, *PRKD3*, *PTH*, *PTPN1*, *RAB7A*, *RAF1*, *RELA*, *RET*, *ROS1*, *RSPO2*, *RSPO3*, *SLC5A5*, *SS18*, *STAT6*, *TAF15*, *TCF12*, *TERT*, *TFE3*, *TFEB*, *TFG*, *THADA*, *TMPRSS2*, *TTF1*, *USP6*, *VCP*, *VGLL2*, *YAP1*, and *YWHAE*) and point mutations in 4 genes (including *BRAF*, *EGFR*, *MET*, *PDGFRA*). All steps were performed according to the manufacturer’s instructions, and the library was sequenced on an Illumina platform as described previously [26].

FISH and RT-PCR methodologies were described in detail in our earlier report [2]. For RT-PCR analysis, we used the primers listed in Table 2.

**Table 2:** Polymerase chain reaction primer descriptions

Gene	CRTC1A	*MAML2B*
Primer mix	Outer: TCGCGCTGCACAATCAGAAG	Outer: GGTCGCTTGCTGTTGGCAGG
	Inner: GAGGTCATGAAGGACCTGAG	Inner: TTGCTGTTGGCAGGAGATAG
RT-PCR	95 °C/14m30s, 35x (95 °C/30 s, 55 °C/30 s, 72 °C/30 s), 72 °C/7 m	95 °C/14m30s, 35x (95 °C/30 s, 60 °C/30 s, 72 °C/30 s), 72 °C/7 m
Primer-specific PCR	95 °C/14 min, 40x (95 °C/1 min, Ann60°C/1 min, 72 °C/1 min), 72 °C/10 min	

#### TruSight oncology 500 kit (TS500)

Mutation analysis and fusion transcript detection were performed using TruSight Oncology 500 Kit (Illumina, San Diego, CA). RNA was extracted using the Maxwell RSC DNA FFPE Kit and the Maxwell RSC Instrument (Promega, Madison, WI) according to the manufacturer’s instructions and quantified using the Qubit HS RNA Assay Kit (Thermo Fisher Scientific, Waltham, MA). DNA was extracted using the QIAsymphony DSP DNA mini (Qiagen, Hilden, Germany) and quantified using the Qubit BR DNA Assay Kit (Thermo Fisher Scientific, Waltham, MA). The quality of DNA was assessed using the FFPE QC kit (Illumina) and the quality of RNA using Agilent RNA ScreenTape Assay (Agilent, Santa Clara, CA). DNA samples with Cq < 5 and RNA samples with DV200 ≥ 20 were used for further analysis. After enzymatic fragmentation of DNA with KAPAFrag Kit (KAPA Biosystems, Wilmington, MA), DNA and RNA libraries were prepared with the TruSight Oncology 500 Kit (Illumina) according to the manufacturer’s protocol. Sequencing was performed on the NovaSeq 6000 sequencer (Illumina) following the manufacturer´s recommendations. Data analysis was performed using the TruSight Oncology 500 v2.2 Local App (Illumina) as described earlier [27].

### Review of the literature

The study intended to examine previously reported cases of SMEC with inflammation. We examined whether SMECs contain typical neoplastic components commonly seen in MEC and whether SMECs demonstrate charcteristic clinicopathological behavior that would require their classification separately from MEC as was proposed for SMECE of the thyroid.

The inclusion criteria for this systematic review were as follows:

1. All research papers must be original research articles that report clinical cases of SMEC.
2. All articles must be published in English.
3. All articles must describe the histologic appearance of SMEC in text and images.
4. All articles must report a confirmation of the diagnosis of MEC using histochemical, immunohistochemical, and/or molecular techniques.

The exclusion criteria were as follows:

1. Studies reviewing previous works without reporting any new cases
2. Studies that investigated non-salivary MECs
3. Studies that published duplicate cases
4. Epidemiological studies and consultation cases
5. Cases published by a predatory journal or non-indexed journal

We conducted an electronic search in PubMed, Science Direct, Web of Science, Scopus, Scielo, Google Scholar, EMBASE (Ovid), Europe PMC, ProQuest, Crossref, and Medline databases. The searched medical subject headings included “mucoepidermoid carcinoma”, AND “salivary gland”, AND “sclerosis*.” The search excluded “thyroid” AND/OR “breast” AND/OR “mammary” AND/OR “lung” AND/OR “pulmonary” AND/OR “pancreas*” AND/OR “skin” AND/OR “cutaneous.”

The time range was customized from 1981 to 2022. Two hundred and eighty articles were found. Duplicated articles were deleted using Mendeley software. There were 88 unique articles. After excluding non-English articles, the number of remaining articles was 53. We then screened the titles and abstracts of all of them and excluded publications not meeting the inclusion criteria. After implementing all the above criteria, the final number of remaining articles was 32 [15–21, 28–52] (the process is described in Supplementary File 1).

## Results

### Clinicopathologic features and follow-up data

The clinical and molecular characteristics of 25 patients with SMEC are summarized in Table 3. There was a female predilection (1.5:1) with a mean age of 44 years (range 16–76 years). A parotid gland mass was the most frequent clinical presentation (23/25; 92%), while minor salivary glands of the palate and the buccal mucosa were affected in one patient each. Macroscopically, the tumors were firm, tan-white to yellow bosselated masses, and some of them had focal cystic change.

**Table 3:** Clinical and molecular characteristics of 25 cases of sclerosing mucoepidermoid carcinomas

No	Sex/age	Site	Size (mm)	Original diagnosis	FISH* MAML2*	FISH *CRTC1*	FISH *CRTC3*	RT-PCR *CRTC1::MAML2*	NGS *CRTC1::MAML2*	Clinical Course (y)	Treatment	Outcome	Follow-up
1	M/26	Paro	17	SPA	neg	ND	ND	ND	neg	Unknown	SPE	NED	3
2	M/55	Paro	40	SMECE	neg	neg	neg	ND	neg	Slowly growing resistance	SPE + RT	NED	4
3	F/47	Paro	12	sclerosing lymphadenoma	+	+	neg	neg	neg	Unknown	RES	Unknown	NA
4	M/19	Paro	10	lymphoepithelial cyst	+	NA	NA	ND	neg	Nodule 4 y	RES	NED	14
5	M/69	Paro	12	sclerosing mucinous cystadenocarcinoma NOS	+	ND	ND	*CRTC1::MAML2*	neg	Slowly growing resistance	SPE	NED	2
6	F/17	Paro	17	Lymphoepithelial lesion or metaplastic WT	ND	ND	ND	*CRTC1::MAML2*	ND	Unknown	SPE	Unknown	NA
7	F/32	Paro	10	SMEC	neg	neg	neg	neg	neg	Excision (positive margins), after 4 mo surgery	LCPE	NED	6
8	F/43	Paro	17	MEC	+	NA	NA	*CRTC1::MAML2*	ND	Local recurrence at 4 y	RPE DLE	NED	14
9	F/25	Paro	50	Obstructive sclerosing sialadenitis	+	+	neg	ND	neg	Unknown	RES	Unknown	NA
10	F/24	Paro	17	SMEC	+	+	neg	*CRTC3::MAML2*	neg	Slowly growing tumor 3 y	LCPE	NED	8
11	F/56	Paro	16	MEC	+	ND	ND	*CRTC1::MAML2*	neg	Excision (positive margins), local recurrence at 2 y	RPE	NED	13
12	M/59	Paro	15	Lymphadenoma or metastatic SCC	ND	ND	ND	ND	ND	Unknown	SPE	Unknown	NA
13	F/49	Paro	30	MEC with reactive fibrosis	+	neg	+	ND	*CRTC3::MAML2*	Unknown	SPE	Unknown	NA
14	F/67	Paro	15	SMEC	+	NA	NA	ND	neg	Excision (positive margins)	SPE	No residual disease DOC	11
15	M/47	Palate	13	SMEC	NA	ND	ND	ND	NA	10 y slowly growing resistance	RES	No residual disease DOC	10
16	M/17	Paro	18	SMEC	+	ND	ND	ND	NA	Unknown	RES	Unknown	NA
17	F/65	Paro	17	SDC	+	ND	ND	ND	ND	Unknown	RES	Unknown	NA
18	M/67	Paro	6	sclerosing lesion/ adenoma	NA	neg	neg	ND	NA	4 y slowly growing nodule	SPE	NED	4
19	M/69	Buccal mucosa	7	Sclerotic lesion	+	+	neg	ND	*CRTC3::MAML2*	Recurrent lesion for 4 y	RES	NED	5
20	F/44	Paro	30	MEC	+	+	neg	ND	*CRTC1::MAML2*	Periglandular LN metastasis RT refused	SPE DLE	NED	4
21	F/62	Paro	30	LESA	+	neg	neg	ND	NA		SPE	NED	10
22	M/76	paro	15	Cystic MEC	+	NA	NA	ND	NA			NED	3
23	F/48	paro	24	SMEC	+	NA	NA	ND	NA			NED	6
24	F/52	paro	15	SMEC	+	NA	NA	ND	NA			NED	5
25	F/32	Paro	10	LG-MEC with TALP	+	ND	ND	*CRTC1::MAML2*	ND		SPE	NED	1

*DLE* dissection lymphadenectomy, *DOC* dead of other cause, *F* female, *FISH* fluorescence in situ hybridization, *LCPE* lateral conservative parotidectomy, *LN* lymph node, *M* male, *MEC* mucoepidermoid carcinoma, *NA* not analyzable/not available, *ND* not done, *NED* no evidence of disease, *Neg* negative, *PE* parotidectomy, *NGS* next-generation sequencing, *Paro* parotid gland, *RT* radiotherapy, *RT-PCR* reverse transcription polymerase chain reaction, *SCC* squamous cell carcinoma, *SDC* salivary duct carcinoma, *SPA* sclerosing polycystic adenoma, *SPE* superficial parotidectomy, *RES* resection, *RPE* radical parotidectomy, *WT* Warthin tumor

All cases were sent as consults, with the correct diagnosis made by referring pathologists in 8 cases. In 5 tumors, the diagnosis was MEC, but the feature of sclerosis as a part of the tumor was neglected in the final report. In 9 cases, the final report considers a broad spectrum of benign tumors and/or lesions, including obscure sclerosing lesions, sclerosing polycystic adenoma (SPA), sclerosing lymphadenoma, lymphoepithelial cyst, benign lymphoepithelial lesion, metaplastic Warthin tumor, and obstructive sclerosing sialadenitis. In 3 cases, another malignancy was speculated, including sclerosing mucinous cystadenocarcinoma NOS, metastatic squamous cell carcinoma, and salivary duct carcinoma, each in one case.

Follow-up data were available for 18 (72%) patients with a mean follow-up of 6.5 years (range 1–14 years). Three patients experienced local recurrences at 2, 4, and 4 years after primary surgery, respectively. One patient had a single periglandular lymph node metastasis and refused radiotherapy. Nevertheless, there was no evidence of disease 4 years after primary diagnosis. None of the 18 patients revealed distant metastasis (Table 3).

### Histological and immunohistochemical findings

**Fig. 1:**
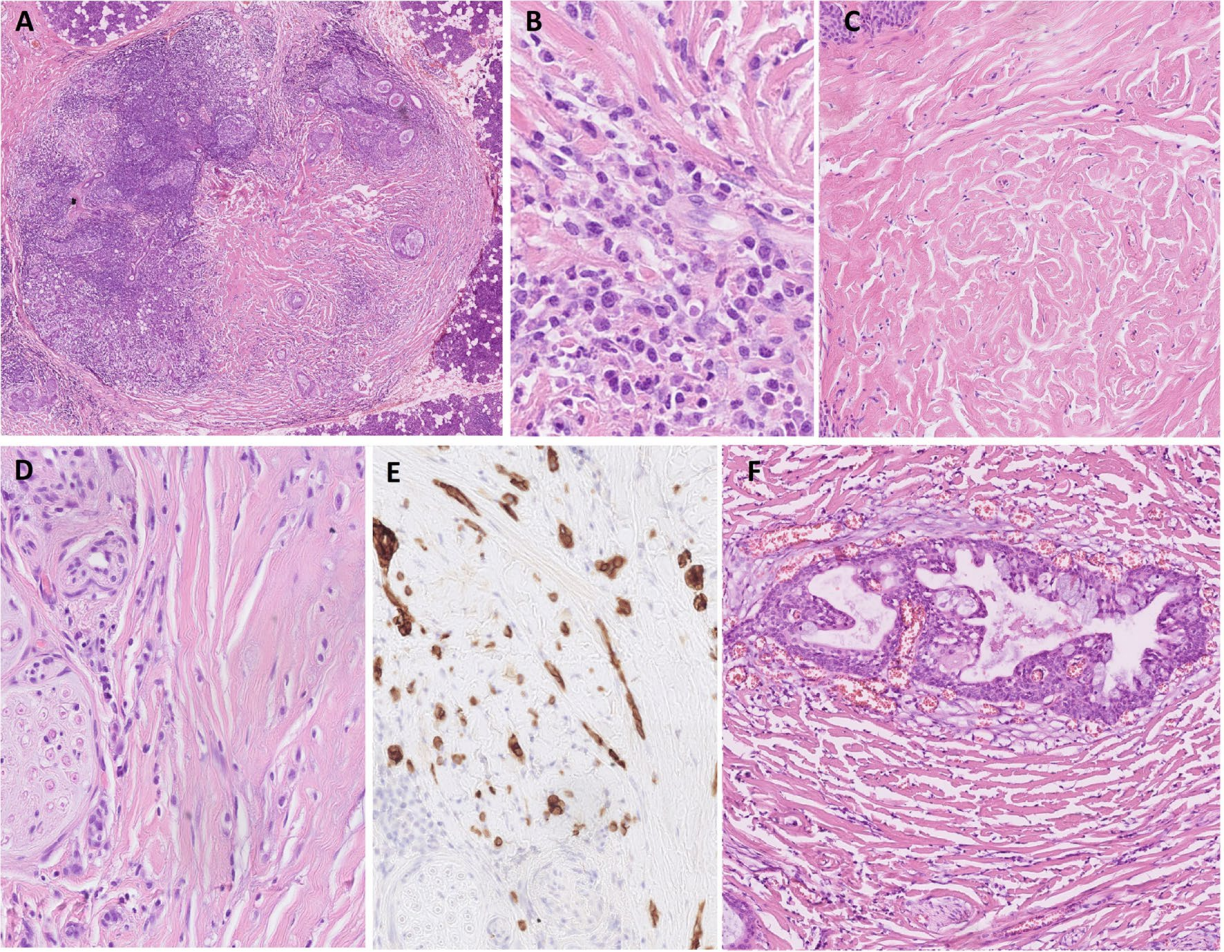
SMECs were well circumscribed and only partially encapsulated by hyalinized pseudocapsule with tumor-associated lymphoid tissue at the periphery including lymphatic follicles with germinal centers and displaying a paucicellular zone of hyalinized collagen in the center **A**. The inflammatory component was mixed, predominantly composed of lymphocytes and plasma cells with only a few cells of acute inflammation (neutrophils/mast cells) **B**. The stromal component was always pronounced and revealed fibrohyaline sclerotic features resembling storiform collagenoma with tumor cells entrapped in a single fashion or as small clusters of cells **C**, **D**. Higher magnification of scattered tumor cells dispersed in collagen bundles **D** and highlighted by AE1/3 **E** on the left of both pictures with entrapped nerve. Occasionally there were characteristic structures of MEC arranged in cystic spaces with intermediate and mucoid cell differentiation **F**

Microscopically, the cases of SMECs without prominent infiltration by eosinophils were well circumscribed and only partially encapsulated by a hyalinized pseudocapsule. At the periphery of the lesion, there was a localized or circumscribed zone of tumor-associated lymphoid tissue (TALP) with occasional lymphoid follicles and even germinal centers (Fig. 1A). TALP was composed of a variable population of inflammatory cells including small and intermediate-sized lymphocytes, plasma cells, histiocytes, and a few neutrophils and/or mast cells. The lymphoid tissue was incorporated in the form of single-cell strips or elongated nests within the lesion and dissected collagen bundles with evidence of retraction artifact at their periphery (Fig. 1B). The tumors were composed of a centrally located paucicellular zone of hyalinized collagen in a scar-like fashion including few fibroblasts (Fig. 1C) and entrapped inflammatory and/or lesional cells which were immunostained by antibodies to cytokeratins AE1/AE3 and CK7 and to p40 and/or p63 (Fig. 1D, E). The stromal component was always pronounced and comprised more than 50% of the total tumor volume and revealed fibrohyaline sclerotic features resembling a storiform collagenoma (Fig. 1C). Tumor cells were less prominent representing 5–20% of the total tumor volume, and they were dispersed in single cell or file fashion between the collagen bundles. Occasionally, the tumor cells grew in variably sized nests or irregular cystic spaces with predominant intermediate cell and mucous cell differentiation (Fig. 1A, F). The tumor cells were spindle-shaped to oval, sharply demarcated, with mild pleomorphism, distinct eosinophilic nucleoli, and a moderate amount of eosinophilic to clear cytoplasm. The mucous cells were distinguished by a cytoplasm distended by a pale mucous vacuole displaying positivity for periodic-acid Schiff and/or mucicarmine. Some cystic structures were compromised, and their mucous content was spilled out into the stroma.

There was only minimal perineural invasion (PNI) in two cases, and occasionally, nerves were entrapped in the hyaline sclerotic stroma (Fig. 1D, E) or at the periphery of the lesion.

Lymphovascular invasion (LVI), necrosis, anaplasia, or prominent mitotic activity were not detected. Proliferative activity was low with mitotic index (MIB1) ranging between 5 and 10%. In 4 cases, the tumors contained foci of a giant cell reaction with multinucleated histiocytes (not shown).

**Fig. 2:**
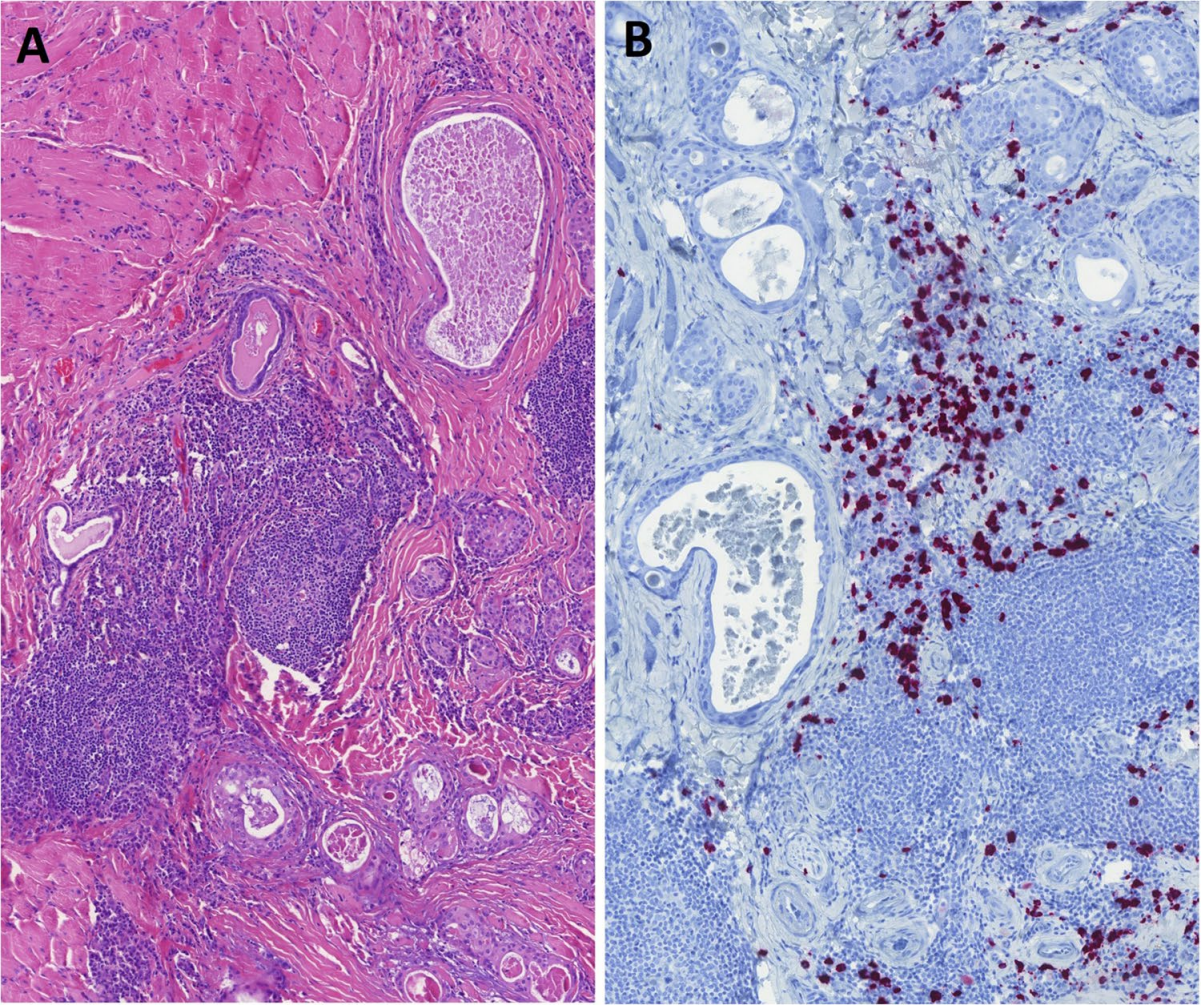
Three cases with typical SMEC-histology characterized by entrapped lesional structures in dense hyalinosclerotic stroma with intense inflammatory (mainly lymphoplasmacytic) infiltrate **A** showed an increased number of IgG4-positive plasma cells **B**

Of these 23 SMECs without eosinophils, 3 cases showed IgG4-positive plasma cells in addition to the classic features of this entity. Immunohistochemical staining shows IgG + and IgG4 + plasma cells in three cases (Fig. 2A, B). The distribution of these cells was non-specifically predominant at the peripheral inflammatory infiltrates with elevated IgG4 + /IgG + ratios (23–33%). However, peri-tumoral areas demonstrate subsidiary cell densities and IgG4 + /IgG + ratios (14–18%). There was no intratumoral detection of either IgG4 + or IgG + cells (Table 4). This may suggest a reactive inflammatory process with increased IgG4-positive cells, yet not meeting strict IgG4-related disease criteria. No other microscopic features of IgG4-related disease, i.e., storiform fibrosis or obliterative phlebitis, were found. None of the patients had systemic IgG4-related disease.

**Table 4:** Quantification of IgG4 + /IgG + plasma cells

Case	Peripheral /hpf		Percent	Peri-tumoral/hpf		Percent	Intratumoral/hpf		Total density
	IgG4 +	IgG +		IgG4 +	IgG +		IgG4 +	IgG +	
1	32	39	23%	2	9	18%	0	0	22.7%
2	19	28	28%	3	10	16%	0	0	24.6%
3	27	36	33%	7	18	14%	0	0	25.7%

**Fig. 3:**
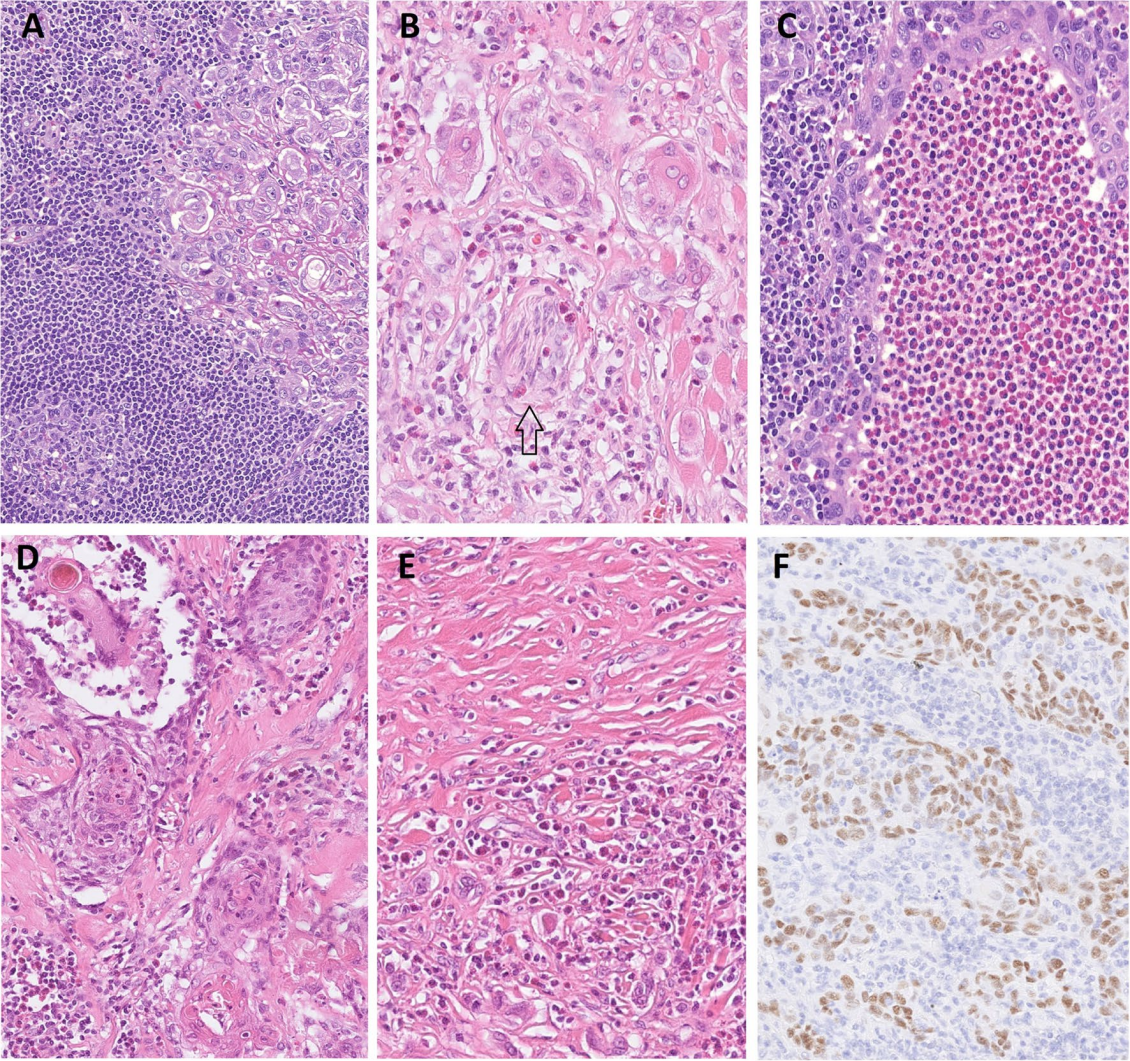
SMECEs were highly cellular with the accumulation of chronic inflammatory cells, in particular lymphocytes that create lymphatic follicles with germinal centers **A**. The lesions were arranged in variably sized nests, thin strands, and anastomosing cords of large polygonal ganglion-like cells; their nuclei were round to oval with prominent nucleoli, vesicular chromatin, and eosinophilic or vacuolized cytoplasm **B** with neural involvement (arrow). Cystic spaces with squamoid cells and evidence of abrupt keratinization **C** and sometimes with multiple eosinophils in their lumen **D**. The stromal component consisted of thin hyalinized membranes and/or fibrous septa that surrounded tumor cells with a retraction phenomenon at their periphery. Occasionally they create larger fibrous fascicles **E**. Tumor cells were positive for p63 **F**

Two SMECs showed a predominant admixture of eosinophils in the inflammatory component (SMECE). These tumors were well circumscribed and almost ovoid lesions with a very thin fibrous pseudocapsule at the periphery (Fig. 3A). The cellularity of the two SMECE cases was high. The neoplastic cells were surrounded and permeated by a dense lymphoplasmacytic infiltrate with an abundant admixture of eosinophils and focal accumulations of chronic inflammatory cells, in particular, lymphocytes in lymphoid follicles with germinal centers (Fig. 3B), and no IgG4-positive plasma cells. The neoplastic cells were haphazardly dispersed throughout the lesion arranged in variably sized nests, thin strands, and anastomosing cords (Fig. 3C). Occasionally, the neoplastic cells created irregular cystic spaces containing PAS-positive amorphous material or eosinophils (Fig. 3D). The neoplastic cells were large and polygonal, almost ganglion-like in morphology, and their nuclei were round to oval with prominent single nucleoli and vesicular chromatin, and the cytoplasm was eosinophilic or vacuolized. Areas of squamoid cells with evidence of abrupt keratinization were present focally (Fig. 3D). The inflammatory cell and neoplastic cell components represented 50% and 30% of the tumor volume, respectively. The stromal component was less prominent when compared to SMEC, and it represented about 20% of the tumor volume. The stroma consisted of thin hyalinized membranes and/or fibrous septa or large fibrous fascicles that surrounded the neoplastic cells (Fig. 3E) positive for p63 (Fig. 3F) and negative for SOX10. In one case, there was PNI (Fig. 3B). None of the SMECs showed LVI, necrosis, or an increased mitotic count. One particular aspect of these tumors is that none of the cases showed both abundant eosinophils and IgG4-positive plasma cells together.

Established grading systems given for MECs were used in all 25 cases [1]. There were 22 and 3 cases graded low- and intermediate-grade, respectively, using the AFIP system, while using the Brandwein modified system, 19, 2, and 4 cases were graded low, intermediate, and high, respectively.

### Molecular profile and fluorescence in situ hybridization

All analyzable cases were studied for *MAML2* gene break or gene fusion using FISH (21/25), RT-PCR (8/25), or NGS (13/25), respectively. Rearrangement of the *MAML2* gene was detected in 18 cases of the 21 analyzable cases tested (86%) by FISH, while this gene locus was intact in 3 cases (14%). In two cases, FISH analysis was not performed (due to lack of tissue material), and two tumors were not analyzable. In addition, six cases of SMECs showed *CRTC1::MAML2* fusion transcript using RT-PCR and three with TS500 NGS methodology, respectively. The used NGS panels investigated cover the fusions and mutations of all genes commonly reported in salivary gland tumors. Only Case#2 showed *PIK3CA*, *NF*, *TCF7L2*, and *PTPRD* mutations. Molecular findings in all 25 cases are summarized in Table 3.

### Review of the literature

Thirty-three articles have reported forty-seven cases of SMEC containing inflammatory infiltrates with one or more cell types (e.g., eosinophils, neutrophils, lymphocytes, and/or plasma cells). Epidemiologically, the reporting countries were the USA (31.9% of the cases), Japan (29.8%), India (14.9%), South Korea (4.3%), and the UK (4.3%). A single case each was contributed from South Africa, Brazil, Canada, Hong Kong, Iran, Ireland, and Romania. Two-thirds of the published cases came from the USA and Japan. See Supplementary file 2 for information on the included cases.

Major salivary glands were the preferable sites of origin of SMEC, including parotid (61.7%), submandibular (14.9%), and sublingual glands (4.3%). In minor salivary glands, SMECs were localized in the palate (6.4%), the parapharyngeal space (4.3%), the retromolar area (4.3%), and the upper lip (4.3%). Females were affected more often than males (2.36: 1). The mean age of patients was 51 years (range 16–81 years). Fifty-seven percent of the reported tumors were small in size (< 2 cm; T1). The remaining cases were medium size (2–4 cm; T2). Many articles reported radiographic measurements of the lesion or macroscopic measures of the gross resection, but radiological information was missing from many case studies (Supplementary file 2).

Low-grade histology was demonstrated in 75% of the reported SMEC cases, while 17% were intermediate-grade, 4% were high-grade, and 4% were not evaluated. In the inflammatory infiltrates, the predominant cells were eosinophils (34%), followed by plasma cells (19%), lymphoplasmacytic cells (17%), neutrophils (13%), lymphocytes (9%), immunoblasts (2%), foamy histiocytes (2%), and mast cells (2%).

The presence of mucin spillage into the stroma forming mucin pools and surrounded by inflammatory infiltrates was observed in all cases. Stromal fibrosis, hyalinization, and sclerosis were also pathognomonic. Other histologic features included cell keratinization (10.6%) [34, 43], eosinophil abscess formation (10.6%) [16, 47], lymph node metastasis (6.4%) [31], tumor-associated lymphoid proliferation (TALP) (6.4%) [18], perineural invasion (4.3%) [29], calcification (4.3%) [17, 30], necrosis (2.1%), apocrine differentiation (hobnail pattern) (2.1%) [47], spindle cell areas (2.1%) [19], pigmentation and dendritic melanocytes (2.1%) [19], and sebaceous differentiation (2.1%) [47]. In 14.9% of the SMEC cases, the inflammatory infiltrates contained also IgG4-positive plasma cells, while storiform fibrosis and obliterative phlebitis were, however, absent [15, 16, 20, 40].

Only ten cases described in the literature were molecularly tested for *MAML2* gene break and/or fusion using FISH or RT-PCR. *MAML2* gene rearrangement was reported in four of these cases (4/10; 40%) [15, 19, 38, 49]. Such a low percentage of MAML2 fusion may be attributed to technical challenges in a tumor with a low number of neoplastic cells.

## Discussion

Mucoepidermoid carcinoma (MEC) is the most common salivary malignancy, and in most cases, it is an easily recognizable tumor. In contrast, the sclerosing variant of salivary MEC (SMEC) with or without eosinophilia is a rare and enigmatic salivary gland neoplasm with a broad differential diagnosis, which presents difficulties in correct categorization. SMEC has a predilection for parotid glands in young/middle-aged patients. It is more commonly encountered in women and usually manifests as a painless slowly growing tumor. Sclerosing mucoepidermoid carcinoma with eosinophilia (SMECE) is an exceptionally rare low-grade variant of thyroid carcinomas. Since its first description in 1991 [53], fewer than 100 cases have been reported [23–25, 54]. The disease presents more frequently in females, on average in the 5th decade. Although some similarities to salivary gland MEC are observed, the current concept is that SMECE of the thyroid is distinct from salivary-type MEC [23, 55]. Histologically, thyroid SMECE shows anastomosing cords and narrow strands of neoplastic mucocytes and epidermoid cells with keratinization and intercellular bridges infiltrating a sclerotic stroma. Thus, thyroid SMECE is morphologically similar but still different from salivary gland SMEC. Unlike the latter, mature squamous differentiation (i.e., keratinization) is a common and even defining feature of thyroid SMECE. Furthermore, the intermediate-type cells that define salivary gland MECs are not present in thyroid SMECE. The stroma in thyroid SMECE reveals an inflammatory background in which prominent eosinophils, lymphocytes, and plasma cells are the main inflammatory cells. Molecular investigation has established that thyroid SMECE is negative for *MAML2* rearrangement [23]. Five cases successfully tested by NGS (ThyroSeq v.2 assay and solid tumor fusion panel) were also negative for mutations and translocations commonly involved in thyroid carcinogenesis [23]. Consequently, thyroid SMECE is not considered to be a part of the spectrum of MEC or papillary thyroid carcinoma, but it is a distinctive entity [22, 23]. In contrast, in our series of salivary SMEC, FISH was more successful in detecting *MAML2* gene rearrangement, and therefore, salivary SMEC seems to represent a rare variant of conventional MEC with the same molecular underpinnings. Although the majority of the reported thyroid SMECE cases had an indolent course [56], aggressive high-grade SMECE with extension to extrathyroid tissues and distant spread have been reported [57]. In contrast, salivary SMEC appears to have a generally favorable outcome.

As shown by the original diagnoses in the present study, the diagnosis of salivary SMEC is challenging because of a broad spectrum of potential diagnostic pitfalls including non-neoplastic inflammatory lesions and benign and/or malignant tumors with prominent hyaline sclerosis. Conventional salivary MEC may contain interspersed fibrous areas usually separating cell nests, while SMECs, with or without inflammatory cells, display prominent sclerosis and keloid-like hyalinized sclerotic stromal foci that may even efface most of the neoplastic proliferation. In our cohort, keloid-like hyaline sclerosis amounted to at least 50% or more of the total tumor volume (mean 63%), regardless of the amount and composition of the inflammatory cells. We assume that this dense sclerosis interferes with the optical properties of the signals the neoplastic cells convey in FISH. Therefore, the greater the volume of the sclerotic area is, the less likely it is that a *MAML2* translocation/rearrangement can be proven. Similarly, NGS analysis might be unsuccessful due to the low number of neoplastic cells in the specimen. This poses additional challenges to the diagnosis of SMEC of salivary glands. Nevertheless, based on above mentioned histomorphological criteria, even the cases either negative or not analyzable for *MAML2* gene break could be classified as highly suspicious for SMEC.

In the differential diagnosis of SMEC, sclerosing polycystic adenoma of salivary glands (SPA) with extensive hyaline sclerotic areas must be taken into consideration [58, 59]. SPA is a benign, often sclerotic tumor that harbors genetic alterations in the PI3K pathway [58, 59] and may show areas of mild to severe dysplasia [59]. The hallmark of SPA is the presence of acini containing large brightly eosinophilic cytoplasmic granules/globules. Although SPA is often associated with dysplasia and most likely represents a precursor lesion of malignancy, it is benign and does not harbor the *CRTC1*::*MAML2* gene fusion [59].

As SMEC cases with abundant IgG4-positive plasma cells were reported [20, 40], IgG4-related disease (IgG4 sialadenitis) enters the differential diagnosis. In our series, we have identified three cases with prominent IgG4-positive plasma cells, but there were not any other features of IgG4-related disease, such as storiform fibrosis or obliterative phlebitis. First, IgG4 sialadenitis typically affects the submandibular glands (usually bilaterally), whereas SMEC most frequently manifests in the parotid gland and as a unilateral tumor. Second, characteristic features of IgG4-related disease, i.e., storiform fibrosis and obliterative phlebitis, are typically not observed in SMEC. Third, any degree of cellular atypia on the fibro-sclero-inflammatory background in salivary glands should raise a suspicion of malignancy (SMEC) as it is typically not seen in IgG4 sialadenitis. More precisely, the presence of “cellular infiltrates suspicious for malignancy” is one of the exclusion criteria for IgG4-related disease [60]. Molecular testing for *MAML2* rearrangement may be of great value in difficult cases. Previously reported features in SMEC such as IgG4-related inflammation, Küttner’s disease, and tumor-associated lymphoid tissue (TALP) cannot be considered pathognomonic for salivary SMEC, because these findings have also been reported in other salivary gland lesions [61]. MEC with inflammation (including TALP), but without sclerosis, is a common finding, but this does not represent SMEC by definition [62].

The current grading systems for mucoepidermoid carcinoma (MEC) are either quantitative (based on scoring) including AFIP [63] and Brandwein [64] or qualitative including a 2-tiered system developed by Xu et al. [65]. Especially when dealing with cases featuring extensive sclerosis, there are discrepancies in how these grading systems assess such cases highlight their deficiencies in offering reliable prognostic information. If the new Memorial Sloan Kettering Cancer Center (MSK) scoring was applied [65], many SMEC cases would be of high grade. Follow-up data from our cases and from the literature indicate, however, that salivary SMECs have favorable outcomes. Established grading systems given for MECs [1] are not reliably applicable to SMEC.

## Conclusion

We reviewed all previously reported cases of salivary SMEC and summarized their shared histologic, molecular, and immunohistochemical findings. In addition, here, we report 25 unpublished cases of SMEC from the consult files of the authors, which is the largest cohort so far. Salivary SMEC is characterized by the formation of keloid-like sclerotic stroma amounting to more than 50% of the tumor volume accompanied by foci of densely packed inflammatory infiltrates subcapsularly or within the neoplasm itself. Such inflammatory infiltrates (eosinophils, plasma cells, or lymphocytes) are usually intermingled with solid nests of neoplastic cells. In contrast to thyroid SMECE which is a distinct entity arising in the background of fibrosing Hashimoto thyroiditis and lacking *MAML2* gene alterations, salivary SMEC is a rare variant of MEC characterized by *MAML2* gene break and/or *CTRC1/CRTC3::MAML2* gene fusion.

## Supplementary Information

Below is the link to the electronic supplementary material.

Supplementary file1 (DOCX 25 KB)

Supplementary file2 (DOCX 30 KB)

## Data Availability

Data supporting the findings of this study are available within the article. The complete datasets generated during and/or analyzed during the current study are available from the corresponding author upon reasonable request.
