# Supplementary material for: Sclerosing mucoepidermoid carcinoma of salivary glands
Source: Virchows Arch. 2024 Nov 14;487(1):33–46. doi: 10.1007/s00428-024-03970-x (PMC12289853; doi:10.1007/s00428-024-03970-x)
Supplement: Supplementary file 1 — Supplementary file1 (DOCX 25 KB) [file 428_2024_3970_MOESM1_ESM.docx]

**Supplementary File 1**. PRISMA flow chart showing the search method

Records identified through database searching
(n = 280)

## Screening

## Included

## Eligibility

## Identification

Additional records identified through other sources
(n = 72)

Records after duplicates removed
(n =88)

Records screened
(n = 88)

Records excluded
(n =35)

Full-text articles assessed for eligibility
(n = 53)

Full-text articles excluded, with reasons
(n = 21)

Studies included in qualitative synthesis
(n = **32**)
