# Supplementary material for: Sclerosing mucoepidermoid carcinoma of salivary glands
Source: Virchows Arch. 2024 Nov 14;487(1):33–46. doi: 10.1007/s00428-024-03970-x (PMC12289853; doi:10.1007/s00428-024-03970-x)
Supplement: Supplementary file 2 — Supplementary file2 (DOCX 30 KB) [file 428_2024_3970_MOESM2_ESM.docx]

**Supplementary Table 2.** Previously reported cases SMEC and their corresponding clinicopathological data

| # | **Authors** | **Year** | **Country** | **Sex** | **Age** | **Site** | **Size** | **Grade** | **IHC marker(s)** | **Imaging** | ***MAML-2*** | **Ref.** |
| --- | --- | --- | --- | --- | --- | --- | --- | --- | --- | --- | --- | --- |
| 1 | Yabuki et al. | 2018 | Japan | F | 73 | Parotid | 2.5× | Low | 1 | CT | Positive | ^15^ |
| 2 | Takashi et al. | 2013 | Japan | M | 79 | Submandibular | 5.6×4.3×3 | Low | ND | ND | Negative | ^16^ |
| 3 | Heptinstall et al. | 2015 | UK | M | 41 | Submandibular | 0.4×1 | Low | 1 | ND | ND | ^17^ |
| 4 | Harada et al. | 2021 | Japan | M | 71 | Parotid | 4 | Low | ND | ND | Positive | ^18^ |
| 5 | Harada et al. | 2021 | Japan | F | 74 | Submandibular | 3 | Low | 3 | ND | Negative | ^18^ |
| 6 | Harada et al. | 2021 | Japan | F | 81 | Palate | 3 | Low | 3 | ND | NA | ^18^ |
| 7 | Harada et al. | 2021 | Japan | F | 60 | Parotid | 3×2.5 | Low | 2 | ND | Negative | ^19^ |
| 8 | Harada et al. | 2021 | Japan | F | 67 | Upper lip | 3.7×1.7 | Low | 5 | ND | Negative | ^19^ |
| 9 | Mendelson et al. | 2010 | Canada | F | 21 | Parotid | 1.2×1.5 | Low | ND | ND | ND | ^20^ |
| 10 | Urano et al. | 2002 | Japan | F | 57 | Parotid | 2.5×2 | Low | ND | ND | NA | ^21^ |
| 11 | Urano et al. | 2002 | Japan | M | 43 | Submandibular | 4.5×2.5 | Low | ND | ND | NA | ^21^ |
| 12 | Chan&Saw | 1987 | Hong Kong | F | 36 | Parotid | 2.2×1.7×1 | Low | ND | ND | ND | ^28^ |
| 13 | Muller et al. | 1997 | USA | F | 17 | Parotid | 2 | INM | ND | ND | ND | ^29^ |
| 14 | Muller et al. | 1997 | USA | F | 60 | Parotid | 2.5 | INM | ND | ND | ND | ^29^ |
| 15 | Sinha et al. | 1999 | Ireland | M | 65 | Parapharyngeal space | 5 | High | ND | ND | ND | ^30^ |
| 16 | Fadare et al. | 2004 | USA | F | 44 | Parotid | 4×2 | Low | 2 | MRI | ND | ^31^ |
| 17 | Ide et al. | 2005 | Japan | M | 28 | Retromolar pad | 2×2 | Low | ND | ND | ND | ^32^ |
| 18 | Heavner et al. | 2006 | USA | F | 23 | Parotid | 2×1 | Low | ND | CT MRI | ND | ^33^ |
| 19 | Kim et al. | 2007 | South Korea | F | 51 | Parotid | 1.4×1 | Low | 3 | 1 | ND | ^34^ |
| 20 | Veras et al. | 2007 | USA | F | 70 | Parotid | 4×3 | Low | ND | ND | NA | ^35^ |
| 21 | Veras et al. | 2007 | USA | M | 37 | Parotid | 2.2 ×1×1 | Low | ND | ND | NA | ^35^ |
| 22 | Veras et al. | 2007 | USA | F | 49 | Parotid | 2.6 ×1.7 | Low | ND | ND | NA | ^35^ |
| 23 | Veras et al. | 2007 | USA | F | 16 | Parotid | 2 | INM | ND | ND | NA | ^35^ |
| 24 | Aguiar et al. | 2008 | Brazil | F | 43 | Parotid | 4×3 | Low | ND | ND | ND | ^36^ |
| 25 | Shinhar et al. | 2010 | India | F | 57 | Parotid | 2 | INM | ND | ND | ND | ^37^ |
| 26 | Kasai et al. | 2011 | Japan | F | 53 | Intraoral | NG | Not graded | ND | ND | Positive | ^38^ |
| 27 | Mardi & Madan | 2012 | India | M | 58 | Submandibular | 2.8×2 | High | ND | ND | ND | ^39^ |
| 28 | Mardi & Madan | 2012 | India | M | 65 | Submandibular | 3×2 | Low | ND | ND | ND | ^39^ |
| 29 | Tian et al. | 2012 | USA | F | 42 | Parotid | 1.5 | Low | *2* | ND | ND | ^40^ |
| 30 | Tian et al. | 2012 | USA | F | 52 | Parotid | 1.4 | Low | 2 | ND | ND | ^40^ |
| 31 | Tian et al. | 2012 | USA | F | 62 | Parotid | 2 | Low | ND | ND | ND | ^40^ |
| 32 | Tian et al. | 2012 | USA | M | 28 | Parotid | 1.2 | Low | *2* | ND | ND | ^40^ |
| 33 | Tian et al. | 2012 | USA | F | 65 | Parotid | 1.5 | Low | ND | ND | ND | ^40^ |
| 34 | Tian et al. | 2012 | USA | M | 32 | Submandibular | 1.1 | Low | ND | ND | ND | ^40^ |
| 35 | Boaz et al. | 2013 | India | F | 73 | Hard palate | 6.5×3.5×2 | Low | ND | CT | ND | ^41^ |
| 37 | Bhat et al. | 2014 | India | M | 32 | Parotid | 4.5×3×2.5 | Not graded | ND | CT | ND | ^42^ |
| 38 | Kinoo et al. | 2014 | South Africa | F | 51 | Esophageal | 3.1 | High | ND | 1 | ND | ^43^ |
| 39 | Lohiya et al. | 2014 | India | F | 45 | Retromolar pad | 4×4 | INM | 8 | CT | ND | ^44^ |
| 40 | Kobayashi et al. | 2015 | Japan | M | 61 | Upper lip | 0.9×1.2 | Low | ND | ND | ND | ^45^ |
| 41 | Bidari-Zerehpoosh et al. | 2016 | Iran | F | 25 | Parotid | 3×3×1.5 | Low | ND | CT | ND | ^46^ |
| 42 | Gherghina | 2016 | Romania | F | 63 | Parotid | 2.7×2 | INM | 6 | US | Positive | ^47^ |
| 43 | Lee et al. **(*)** | 2017 | South Korea | F | 69 | Sublingual | 3.7 | Low | ND | CT | ND | ^48^ |
| 44 | Sato et al. | 2017 | Japan | F | 76 | Sublingual | 2 | Low | 2 | CT MRI | Positive | ^49^ |
| 45 | Devi et al. | 2018 | India | F | 35 | Parotid | 4×5 | INM | ND | CT | ND | ^50^ |
| 46 | Fujioka et al. | 2020 | Japan | M | 27 | Parotid | 4 | Low | ND | CT | ND | ^51^ |
| 47 | Rasul et al. | 2020 | UK | F | 23 | Parotid | 2.5×1 | Low | ND | MRI | ND | ^52^ |

**CT**: Computed tomography; **INM**: intermediate ; **MRI**: Magnetic Resonance Imaging; **NA**: Not analyzable; **ND**: Not done; **OPT**: Orthopantomography; **US**: Ultrasonography (Superficial).

**(*):** The same manuscript was published twice (an original in English and a French translation)
